# First Attempt to Infer Sound Hearing and Its Paleoenvironmental Implications in the Extinct Insular Canid *Cynotherium sardous* Studiati, 1857 (Sardinia, Italy)

**DOI:** 10.3390/ani12070833

**Published:** 2022-03-25

**Authors:** Marco Zedda, Antonio Brunetti, Maria Rita Palombo

**Affiliations:** 1 Department of Veterinary Medicine, University of Sassari, 07100 Sassari, Italy; 2 Department of Biomedical Sciences, University of Sassari, 07100 Sassari, Italy; brunetti@uniss.it; 3 CNR-IGAG c/o Department of Earth Sciences, Sapienza University, 00185 Rome, Italy; mariarita.palombo46@gmail.com

**Keywords:** *Cynotherium sardous*, Medusa-Dragonara cave, Sardinia, Late Pleistocene, petrosal bone, cochlea, earing capability, echolocalization, island rule, paleoenvironment, Carnivora

## Abstract

**Simple Summary:**

The microtomographic approach allows nondestructive acquisition of anatomical details of the bone labyrinth that houses the inner ear. The petrosal bone can be a gold mine of information for a variety of questions in different research fields, including taxonomic, behavioral, and genetic studies. The semicircular canals provide information on head posture and locomotor ability, whereas the cochlea provides data on hearing ability. The petrosal bone is the hardest structure in the skeleton and could be well preserved in fossil specimens. As a result, it is becoming more and more popular in current archaeological and paleontological studies. In this study, petrosal microtomographic analysis was applied for the first time to *Cynotherium sardous*, a highly modified endemic canid that inhabited Sardinia during the Middle to Late Pleistocene. Indications about its hearing ability may provide interesting insights to better understand the new lifestyle and behavior this canid acquired during the long evolutionary process it underwent in the peculiar insular ecosystem with a depleted fauna. The poor hearing and echolocalization capabilities of *Cynotherium sardous* would have been the outcome of reduced competition pressure due to the absence of predators and the abundance of prey, such as the large ochotonid *Prolagus sardus,* while the high-frequency hearing could be interpreted as an adaptation to detect sounds emitted by its preferred prey.

**Abstract:**

This is the first study on the bony labyrinth of *Cynotherium sardous*, an intriguing extinct canid that inhabited Sardinia in the late Middle and Late Pleistocene. The morphological features of the cochlea indicate that *C. sardous* had a lower number of cochlear turns (2.25) than all extant canids. This feature, as well as the reduced length of the spiral canal, the cochlear curvature rate, and the narrow basal membrane, indicates that *C. sardous* had poor hearing abilities limited to high-frequency sounds with a low limit of 250 Hz and poor echolocalization skills. From the data available, it is not possible to infer whether *C. sardous* was unable to echolocalize its prey and relied on other senses (e.g., smell and sight) to locate them or whether the acoustic range of *C. sardous* was specialized for identifying the sounds produced by its most common prey to transmit signals for predator warnings or group communication. All things considered, the results obtained confirm the utility of cochlea morphological studies in reconstructing the hearing abilities of this species and in providing some suggestions about its ethology, but they fall short of providing any new sound evidence regarding the ecological role of *C. sardous* in the Late Pleistocene Sardinian ecosystem.

## 1. Introduction

The shape of the bone labyrinth, the anatomical system that accounts for equilibrium and hearing, may provide some insights into the function, ecology, and phylogeny of extant and extinct mammalian species. However, for the majority of the 20th century, research and data on mammalian inner ears mostly concerned extant animals.

Studies on the fossil inner ears of prehistoric mammals have long been uncommon as they mostly rely on anatomical sectioning and microdissection techniques, which require specimen destruction and is impractical if only one or a few remains are available. The adoption of contemporary nondestructive techniques, such as microtomography (µ-CT), which produces high-resolution 3D pictures, has allowed considerable expansion of knowledge and comparative research on the anatomy of the inner ear in various animal species in recent decades [1,2].

The bony structures (bony labyrinth) of the inner ears are housed in the temporal petrous bone, which is the skeleton’s densest bone [3], providing exceptional protection to the structure. The cochlea, which is involved in hearing, forms the anterior part of the bony labyrinth, the bony vestibule forms the median part, and the three semicircular canals involved in the body equilibrium form the posterior part of the bony labyrinth. The vestibule connects the cochlea and the semicircular canals and communicates with the tympanic cavity via the vestibular window [4].

The anatomy of the bony labyrinth is generally conservative in vertebrates. It could be relevant in morphofunctional studies and could provide some information to solve phylogenetic and paleobiological problems that have mainly been scrutinized by studying teeth [5], although the cochlea provides more phylogenetic signals than the semicircular canals [6,7,8,9]. Indeed, mechanosensory organs are useful anatomical systems to learn about the function, ecology, and phylogeny of living and extinct vertebrates [9]. The vestibular system regulates the sense of balance and spatial orientation in movement coordination, while the semicircular canals (sensory detectors of head rotational acceleration and their mutual degree of orthogonality) may provide useful clues for inferring agility and head angular velocity during locomotion [7,10,11,12,13,14]. Furthermore, various studies have attempted to establish a link between the morphology of the cochlea and the hearing ability of mammals.

In particular, anatomical studies of the inner ear are highly suitable for establishing correlations between behavior and anatomy [7,11,12,14,15,16,17,18]. For instance, interesting correlations between morphological characteristics of the inner ear, including the cochlea, and the aquatic or terrestrial lifestyle of individual species were found after analyzing 35 species of living mustelids [7]. Several studies have indicated that the cochlea, specialized for sound detection, shows morphological differences within vertebrates that are useful for phylogenetic correlations [13], and several studies have attempted to clarify the correlation between cochlea morphology and hearing.

Studying the labyrinth of extinct endemic mammals has important implications from phylogenetic, paleobiological, and ecological points of view. This type of research could be particularly interesting when applied to extinct insular mammals because most of them modified their size, morphofunctional traits, and life behavior, thus resulting in extreme and fascinating species whose origins are often difficult to determine.

The aim of this research was twofold: (i) to describe for the first time the morphological features of the bony labyrinth of *Cynotherium sardous*, an endemic canid that lived in the Corso-Sardinian Massif during the late Middle and Late Pleistocene and (ii) to infer its hearing ability based on the peculiar insular environments it inhabited.

### Cynotherium sardous

Carnivoran predators are uncommon on islands, and some authors believe that their absence or scarcity is the primary cause of size variations (“dwarfism” and “gigantism”) among insular animals. Extant and fossil terrestrial and semiaquatic Carnivora, on the other hand, have been found in more or less impoverished and disharmonic endemic faunas on continental islands, while they are uncommon on oceanic and oceanic-like islands. The Pleistocene Sardinian faunas include the highest number of endemic carnivoran species and the only dwarfed top predators thus far recorded in Cenozoic oceanic-like islands, together with four otters, two mustelids, and the dwarfed medium-sized canid *Cynotherium sardous* Studiati, 1857, which is the best known endemic carnivoran from the Late Pleistocene deposits of the Corso-Sardinian Massif (e.g., [19,20] and references therein).

The difficulty in studying this canid, as with most endemic insular endemic species, is that the morphological traits of *C. sardous* have been highly modified in comparison to its mainland ancestor during the evolutionary process that *Cynotherium* underwent in the new insular ecosystem. Indeed, *C. sardous* displays a peculiar mixture of craniodental features, some of which are thought to be related to hypercarnivory (e.g., unicuspid talonid on m1), while others (such as the reduced sagittal crest and the long temporal lines) have been hypothesized to be related to pedomorphosis [21]. As a result, it is difficult to separate its plesiomorphic characters, which it inherited from its mainland parent, from its apomorphic characteristics, which it acquired during its evolution in the strange insular environment. As a result, the systematic position of *Cynotherium* and its evolutionary relationships remain unresolved questions.

Initially thought to have stemmed from *Cuon* due to the presence of a single cuspid in its lower carnassial tooth (see [22] for a discussion), representatives of the *Cynotherium* lineage (*Cynotherium* sp., *Cynotherium malatestai*, and *C. sardous*) were successfully considered by most authors to be descendants of one of the Early Pleistocene dogs (*Canis etruscus, Canis arnensis*, and *Xenocyon falconeri* or *Xenocyon lycaonoides*) [20,22,23,24,25,26,27,28]. Some years ago, new dentognathic remains of a large canid were found at Grotta dei Fiori (central western Sardinia) in deposits older than 500 ka [29] and ascribed to the new species *C. malatestai.* The morphological features of the lower dentition of the new species, particularly the marked hypercarnivorous character of carnassial, support the hypothesis of *Cynotherium*’s origin being from the late Early Pleistocene population of *X. lycaonoides* [30].

Recently, a genomic study was conducted on specimens from Corbeddu cave [31], representing the most recent *C. sardous* remains known thus far (11,405–11,199 BC) [32]. The results obtained by the research team confirm, on the one hand, that the *Cynotherium* insular canid lineage is distinct from all other living canids from Eurasia, Africa, and North America and, on the other hand, that it is genetically linked to the Asian wild dog (*Cuon alpinus*) and diverged from the dhole lineage around 885 ka. The dhole is a middle-sized hypercarnivore canid that lacks the last lower molar and has a specialized lower carnassial [33]. The presence of the last lower molar in *C. malatestai* and *C. sardous* [22] appears to be at odds with genomic data. *Cuon* is first recorded in Southern Europe at l’Escale (France), a site believed to date to the beginning of the Middle Pleistocene (0.73–0.6 Ma; MIS 17) [34], while *Cuon alpinus* is mainly reported from a few Late Pleistocene sites in Italy. The supposed origin of *Cynotherium* from *Cuon* and the hypothesized date of the divergence of the continental and insular lineages have important implications regarding the time of ancestor dispersal to the Corso-Sardinian Massif and the evolutionary changes undergone by the insular lineage. However, discussing such implications goes well beyond the purposes of our study.

Whatever the actual taxonomic position of *Cynotherium* might be, some dental micro-features and skeletal morphofunctional traits enable us to provide some hints about its role within the Late Pleistocene Sardinian mammalian community.

Cranial and dental anatomy, occlusal mechanics, area of origin and insertion of the main muscles involved in the mastication process, bite force, lower molar grinding area, tooth enamel structure (Hunter–Schreger band (HBS) from undulating to angulate in premolars), and the microscopic defects (microwear) produced during the food masticatory process suggest a predominant faunivory habit, occasionally including a very small amount of vegetables, whereas the presence of a zig-zag pattern in the talonid of the lower carnassial and second lower molar indicates the aptitude to break gracile, small bones [35,36,37].

In addition, *C. sardous* shares several basic morphological features with other canids but shows some peculiar characteristics of the origination/insertion region of some muscles (e.g., the muscles obliquus capitis cranialis and caudalis, splenius, omotransversarius, deltoideus, teres minor, triceps, and anconeus) of the mastoid cranial region, atlas, scapula, humerus, and ulna, which suggest that *Cynotherium* had a powerful flexion of the shoulder and extension of the elbow, could rotate the neck and flex the head downwards much more than other extant canids, and was capable of a powerful flexion of the shoulder and extension of the elbow (cfr. [27] for more details).

These peculiar characteristics point to a stalking hunter behavior specialized on the large lagomorph *Prolagus sardus* [27], a hypothesis supported by the feeding behavior of *C. sardous* [35,36].

## 2. Materials and Methods

### 2.1. Materials

**Figure 1:**
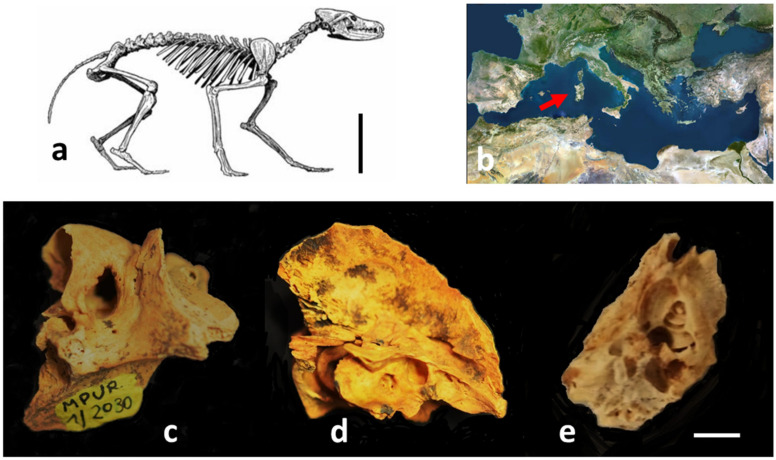
(**a**) Skeleton of *Cynotherium sardous* (Reprinted with permission from Ref. [22]. 1970 Mem. Ist. It. Paleontol Um, modified), scale bar = 20 cm; (**b**) the arrow indicates the island of Sardinia in the Mediterranean Sea; (**c**–**e**) portion of temporal bone in lateral view (**c**), medial view (**d**), and after microdissection (**e**), scale bar = 3 mm.

Our analysis was performed on an isolated petrosal bone (inventory number MPUR 1/2030) currently included in the paleontological Malatesta collection, stored at the Museum of Earth Sciences (MURST) (Department of Earth Sciences, Sapienza University, Rome, Italy). The specimen belongs to the rich *Cynotherium sardous* sample from the Medusa-Dragonara cave (Porto Conte Bay, Alghero) (Figure 1). The fossil deposit, dated to the Last Glacial Maximum (LGM) [38], was discovered in the late 60s by Professor A. Malatesta, who extensively described and illustrated the fossil canid remains [22].

### 2.2. Methods

To analyze morphological aspects of the bony labyrinth of *C. sardous* and collect morphometrical data relevant to comparing them to what is reported in the literature on current and extinct canids, we carried out an anatomical microdissection and µ-CT imaging of the petrosal bone.

#### 2.2.1. Microtomography

We performed tomographic measurements using a 1172 Skyscan tomograph (Bruker) working at 100 kV and 100 µA and acquired a set of angular radiographic images, also known as projections, from a range of angles (every 0.2°) all around the sample. A total of 1800 projections were acquired. Each measurement lasted approximately 2 h. The spatial resolution was 4.6 µm. The acquired data were reconstructed using the Skyscan software based on GPU cards and then visualized using the CTVox software (Bruker). For CT scanning, the X-ray beam was filtered with a 0.5 mm thick aluminum filter, and the reconstruction software used a Hamming filter with alpha = 0.54. Unfortunately, the semicircular canals were likely obliterated during fossilization by diagenetic processes, so our study had to be limited to the cochlea.

#### 2.2.2. Morphometry

The following parameters were recorded using the software Analyze 12.0 (AnalyzeDirect, Overland Park, KS, USA): cochlear width (Cow) = maximum diameter of the basal turn of the spiral canal; cochlear height (Coh) = distance between the plane tangent to the basal turn of the spiral canal and the cochlear apex; number of the turns of spiral canal (Con), numbered from the basal turn to the cochlear apex; length of the spiral canal (Col), imaging the cochlea unrolled; radius of the basal turn (R_basal_); radius of the apical turn (R_apex_); ratio between radius of the basal turn and radius of the apical turn (ρ = R_basal_/R_apex_); width of the spiral canals at different turns (Scw); and width of the basal membrane (Bmw) at level of the basal turn, measured between the edge of the osseous spiral lamina and the secondary lamina. All the parameters are indicated with the abbreviations proposed in two works [5,39].

## 3. Results

**Figure 2:**
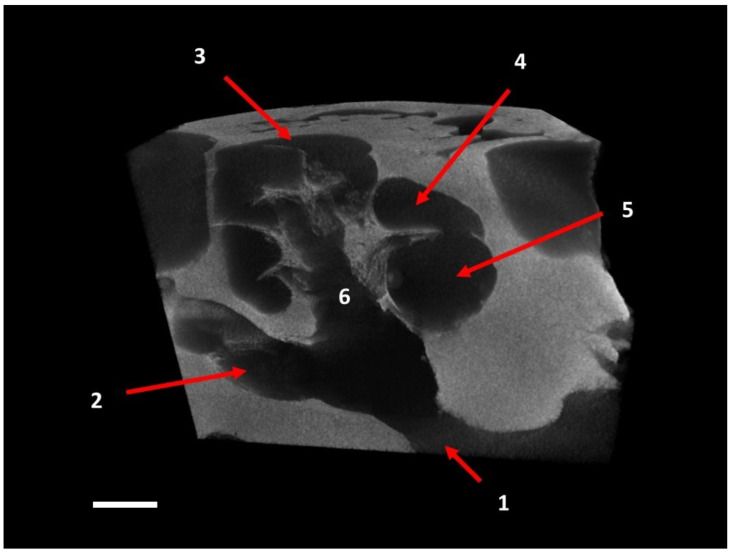
Vertical cross section of the cochlea at level of the modiolus. 1, internal acoustic meatus; 2, beginning of the scala tympani near the round window; 3, last turn; 4, scala vestibuli; 5, scala tympani; 6, bony cavity of the modiolus housing the cochlear nerve (3D reconstruction by µ-CT projections). Scale bar = 1 mm.

**Figure 3:**
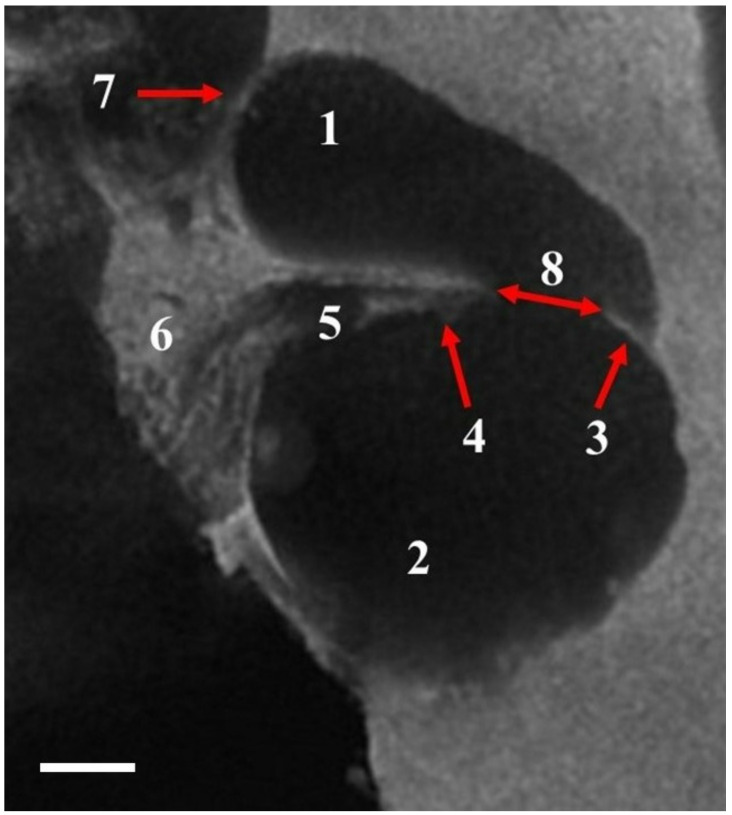
Cross section of the spiral cochlear canal at level of the basal turn. 1, scala vestibuli; 2, scala tympani; 3, secondary spiral lamina (only in the basal turn); 4, osseous spiral lamina; 5, spiral cribriform tract containing the spiral ganglion; 6, modiolus; 7, lamina of modiolus; 8, space containing the basal membrane. Scale bar = 400 µm.

The analysis of 3D images, as well as the anatomical microdissection of the dorsal wall of the petrosal bone, allowed detailed reconstruction of the cochlear morphological features. Even the tiniest and most fragile bone structures were well preserved and easily identifiable. The cochlea had a rounded apex and was conical in shape. The spiral canal surrounding the modiolus formed 2.25 turns. The cone’s base was 4.7 mm wide, which was larger than the entire height (4.1 mm). The transversal part of the spiral canal was virtually circular, and its diameter decreased gradually from the tympanic window to the apex. The width of the turns was 1.6 mm in the first and 0.8 mm in the second turn, and the apex was 0.3 mm wide. The cochlea had the shape of a logarithmic spiral, and the length of the outstretched spiral canal was 25.8 mm owing to the concentric arrangement of the coils. Scala vestibuli and scala tympani were easily distinguished in the spiral canal due to the presence of a robust osseous spiral lamina that separated them (Figure 2 and Figure 3).

A secondary spiral lamina was present in the exterior wall at the level of the basal turn and was easily discernible in µ-CT images as a thin radiopaque line (Figure 3). The basal membrane, which was 0.4 mm wide in the basal turn, filled the space between the free edge of the osseous spiral lamina and the secondary spiral lamina, which were placed in the internal and exterior walls of the spiral canal, respectively (Figure 3). The width of 0.4 mm was only about 1/4 of the 1.6 mm width of the spiral canal. The radius of the spiral canal decreased from the first turn (2.35 mm) to the second (1.8 mm) and at the apex (0.7 mm). The resulting ratio of cochlear curvature (ρ = R_base_/R_apex_) was 3.35. A comparison between the osteometric data of *C. sardus* cochlea and the values obtained by averaging the measurements taken on cochleas belonging to various carnivore species [5] and 92 canids [40] highlighted the peculiarity of the Sardinian canid (Table 1).

**Figure 4:**
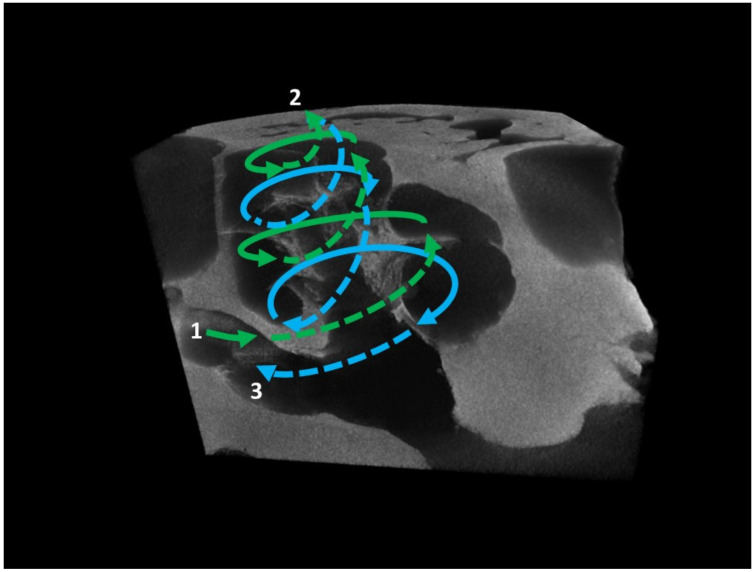
Reconstruction of the direction of the pressure wave in the perilymphatic liquid in the scala vestibuli (green) and in the scala tympani (blue). 1, beginning of the scala vestibuli, near the oval window; 2, helicotrema, point in the apex of the cochlea where both the scalae communicate; 3, beginning of the scala tympani near the round window.

**Figure 5:**
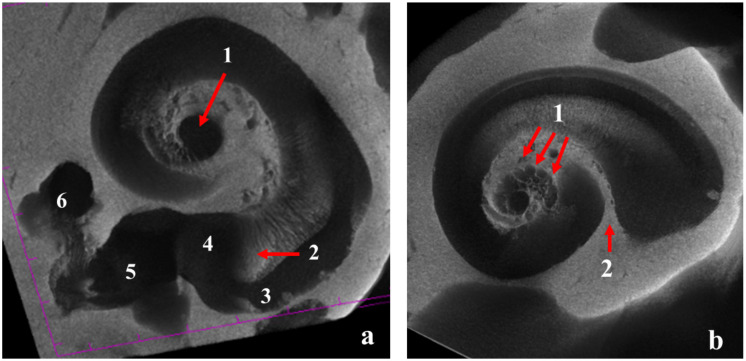
Horizontal cross sections of the cochlea at different levels of the spiral canal. (**a**) Basal turn: 1, modiolus with the bony cavity housing the cochlear nerve; 2, beginning of the osseous spiral lamina; 3, beginning of the scala tympani near the round window; 4, beginning of the scala vestibuli near the oval window; 5, vestibular cavity; 6, ampullar portion of semicircular canals. (**b**) Last turn: 1, cavities housing the spiral ganglion; 2, lamina of modiolus separating the first from the second turn of the cochlear canal.

The modiolus had a conical shape and was surrounded by a bony wall with multiple cavities holding cochlear nerve fibers that converged in the central hollow bony core of the modiolus. Within the vestibule, the osseous spiral lamina began and finished at the apex of the modiolus (Figure 4). Numerous modiolar canals were seen on both the wall of the modiolus and the osseous spiral lamina, which were meant to accommodate blood vessels, nerve fibers, and ganglion cells of the cochlear nerve (Figure 5). The beginning of the scala vestibuli and the scala tympani, as well as the vestibulum cavity and ampullar portions of the semicircular canals, were also recognizable in some µ-CT 3D sections (Figure 5).

## 4. Discussion

A cochlea is the part of inner ear specialized in sound detection, and several studies have attempted to determine the relationship between the cochlear morphology and the hearing abilities of a species. Cochlear morphology differs in different lineages and can help infer phylogenetic relationships [13]. For example, coiling is absent in reptiles, birds, and monotreme mammals, and it likely evolved in marsupials and placental mammals [16] to fit a long organ into a tiny space inside the petrosal bone. The cochlear shape, especially the length and number of coils, which are related to the hearing frequency, may represent a valid phylogenetic signal [41,42,43]. For instance, they vary from less than one turn in Monotremes to approximately five turns in Caviomorpha [1,15,17,42,43,44]. Phylogenetic relationships based on cochlear and inner ear features have been inferred in some taxonomical groups, such as primates [10,45,46,47,48] and extinct deer [13].

Furthermore, an animal’s sensitivity to acoustic frequencies varies with the length and number of turns of the cochlear spiral, so the loudness of a frequency heard by an individual can be inferred from its cochlear length and turn number. In particular, a high number of turns characterizes mammals with sensitivity to low-frequency sounds. Elephants and cows, for example, have a high number of turns, suggesting they are sensitive to low-frequency sounds [49], while a scheme predicting primate hearing sensitivity was developed by analyzing the length and the turn number (ranging from 1 + 7/8 to 3 + 1/8) of the cochlea in 21 extinct species [50]. Accordingly, the auditory abilities of extinct animals can be deduced from their fossil petrous bones [51]. Given that the perception of high- and low-frequency stimuli (possibly collected near the oval window and at the end of the cochlear spiral) is related to the development [39] and curvature degree of the cochlear spiral [49], the hearing frequency in extinct mammals may be predicted in well-preserved fossil cochleas by the ratio of the apical and basal radius turns [39,41]. The perception of the sound frequency also depends on the basilar membrane rigidity, which in turn is related to its dimensions; a narrow basilar membrane is more rigid than a wide one [49]. In fossil specimens, the membrane dimension can be estimated by the distance between the primary and secondary bony spiral laminae (named “basilar gap” or “laminar gap” by some authors, e.g., [52,53]). Moreover, basal membrane features could provide some hints about phylogenetic relationships [6,52,53,54,55,56].

### 4.1. Hearing Ability in Cynotherium sardous

The cochlea and tympanic cavity of *C. sardous* are similar to those of Carnivora, but the number of cochlear turns in *C. sardous* (only 2.25) is definitely lower than the number known in other carnivore species. For instance, there are 3.5 cochlear turns in wolves (*Canis lupus*) [40,57], about 3 in North American *Hyaenodon* [58], 3–3.3 in the domestic cat (*Felis catus*) [4,59,60], and 2.5 in European *Hyaenodon exiguus* [5]. In domestic dogs (*Canis familiaris*), the number of turns is variable, ranging from 3.0 [40] to 3.25 [4] and 3.3 [60] up to 3.75 [1], while wolves have about 3.2 turns on average, i.e., about 1/3 of a turn less [57].

The cochlear dimensions of *C. sardous* are roughly similar to those of other Carnivora. In particular, the height (4.1 mm) is slightly higher than the average medium value (3.71 mm) but falls in the range described for 19 carnivore species (Table 1) [5]. Conversely, the *C. sardous* cochlea width (4.7 mm) is smaller than those of all 19 of the same species (average width = 7.1 mm), and the outstretched length is lower than that of 17 of the species described by [5], with only two species, *Hyaenodon* (22.4 mm) and *Paguma larvata* (25.7 mm), having shorter cochlea (Table 1).

Bringing together these data, the cochlea of *C. sardous* appears to be small and underdeveloped. According to [39], a spiral-shaped cochlear may improve sensitivity to low-frequency sounds by redistributing wave energy towards the outer wall, particularly along its innermost, tightest, apical turn, and enhancing low-frequency hearing. Low-frequency sensitivity probably depends on a stepwise reduction of the turn diameter from the basal to the apical one [49]. When compared to other vertebrates, such as *Felis catus* (ρ = 6.2), *Bos taurus* (ρ = 8.9), *Elephas maximus* (ρ = 8.8), and *Chinchilla lanigera* (ρ = 6.4), the ratio between the radius of basal and apical turns is quite low in *C. sardous* (ρ = 3.35), whereas it is quite high when compared to *Mus musculus* (ρ = 1.7) and *Rattus norvegicus* (ρ = 3.1) [41] These data and the position of *C. sardous* in the diagram showing the low-frequency hearing relationship with spiral radii ratio in some land and aquatic mammals reported by [41] suggest that the hearing capability in *C. sardous* was likely oriented towards high frequencies because its low-frequency limit of hearing was about 250 Hz. A comparison between the rigidity of the *C. sardous* membrane and that of other canids is unfortunately impossible because no measurements of the distance between the primary and secondary bony spiral in canids were found in the literature.

Considering that the known cochleas of extant and extinct carnivores have a medium number of more than 3 turns, *C. sardous*, which has 0.75 (3/4 of a turn) turn less, is the species with the least number of turns among carnivores. However, the low number of cochlear turns of *Cynotherium* could hardly be related to phylogeny because it is significantly lower than the minimum value in the narrow variation range of wild canids. On the other hand, the number of turns does not depend on the dwarfed size of *Cynotherium* because it is not the number of turns but rather the length of the cochlear canal that is positively related to body mass [6,43,49]. Therefore, it seems reasonable to suppose that the low number of cochlear turns could be related to the possibility of hearing a small range of frequencies. Furthermore, given the role of the basal membrane in sound propagation and the fact that the dimension of the basal membrane is related to the window width (larger, more elastic basal membranes account for low- and high-frequency sounds), the reduced width of the basal membrane observed in *C. sardous* suggests a specialization for hearing high-frequency sounds. In fact, the tympanic membrane transmits external sounds to the cochlea via the three small bones that connect the tympanic membrane to a second membrane inserted into the vestibular, oval window. Cochlear fluids seal one of the three chambers of the fluid-filled cochlear spiral. The resulting pressure swings in cochlear fluids deflect the basilar membrane and spans the length of the spiral cochlea. This deviation propagates along the basal membrane from the oval window towards the apex of the spiral canal [41].

Overall, the cochlear features of *C. sardous* indicate that it had poor hearing abilities, likely limited to high-frequency sounds.

Another fascinating aspect of the hearing of *C. sardous* is its poor echolocalization ability. It is known that there is a significant delay (interaural time difference) between the arrival of a sound in one ear and the other. Animals can detect the source of the sound thanks to this time lapse. Because this echolocalization capability appears to be more efficient with low-frequency sound than with high-frequency sound [61], we can assume that *C. sardous* was unable to echolocalize its prey and had to rely on other senses to locate them (e.g., smell and sight).

### 4.2. Inferences about Evolutionary Processes of Cynotherium in the Sardinian Insular Environment

Large mammals in the distinctive insular habitat, characterized by low biodiversity, ecologically unbalanced fauna, and low intraspecific competition pressure, are well known to undergo rapid evolutionary processes that can take thousands or hundreds of years to happen [62,63]. These processes generally include not only changes in size and proportions (the graded phenomenon known as the “island rule” [64,65]) but also structural and morphofunctional changes that could be simply related to changes in the distribution of body mass load or to the new way of life adopted by island dwellers in their new insular environment. The modifications may be so significant in comparison to the mainland progenitor that they obscure the evolutionary links of endemic species. The alterations primarily affect the musculoskeletal system and teeth, but they also affect the visual and olfactory systems, particularly in artiodactyls. For instance, these changes have been observed in the highly modified bovid *Myotragus balearicus* and in the smallest Cretan endemic deer *Candiacervus ropalophorus* and *Candiacervus* spp. II (sensu [66] but including three species, *Candiacervus devosi*, *Candiacervus listeria*, and *Candiacervus reumeri*), which show small orbits, small olfactory bulbs, and reduced visual brain structures. Moreover, the sense-dependent centers of olfactory, acoustic, and optic functions are especially small in the *Myotragus* brain compared to those of the extant wild small-sized bovids ([67] and references therein). These changes parallel those shown by domesticated animals and have been interpreted as an adaptive response to insular environmental conditions, especially to lack of interspecific competitors and large predators.

The peculiar modifications undergone by Cenozoic insular taxa have been observed not only in Mesozoic dinosaurs, e.g., in the dwarf sauropods *Europasaurus holgeri* (Kimmeridgian, Germany [68]) and *Magyarosaurus dacus* (Maastrichtian, Romania and Hungary [69]), but also in the small primitive monotreme *Litovoi tholocephalos* from the latest Cretaceous of Romania. The latter species shows a reduction in brain size and heightened sensory acuity compared to its continental relatives. These apomorphies suggest it lived in an isolated environment despite the lack of a remarkable change in body size [70]. Therefore, it seems that modifications in sensorial organs and function have occurred in insular mammals since the beginning of their evolutionary history.

Changes in brain size and the sensorial system have been reported in some but not all insular–dwelling mammals. The few studies on proportional changes in the encephalon volume of insular endemic mammals (including *Homo*) report, for example, a reduction in the dwarf Florens hominin *Homo floresiensis* [71], in the Balearic bovid *M. balearicus* [72], and in the dwarf hippopotamus from Madagascar *Hippopotamus madagascariensis* [73] (but cf. [74]). However, no significant changes were observed in the smallest Cretan deer [67]. Conversely, dwarf straight-tusked elephants show an increase in relative brain volume proportional to their size reduction [74], reaching a maximum in the smallest Sicilian elephant *Palaeoloxodon* ex gr. *P. falconeri* [75]. Variations in brain size or significant anatomical brain modifications have not been reported to date in insular predators. For instance, among insular otters, the Sicilian *Lutraeximia trinacriae* [76] and the Sardinian *Sardolutra ichnusae* [77] show an expansion of the coronal gyrus (corresponding to the somatic sensory area), implying remarkable sensitivity of the facial vibrissae as in mainland *Lutra*.

Available data suggest that the insular environmental conditions did not induce any important brain changes in the endemic predator *C. sardous*. In particular, no brain size reduction has been detected in specimens from the Dragonara-Medusa cave [78] and in an individual from the Corbeddu cave [26,27], both of which have brain sizes comparable to those of living mainland canids of comparable body mass. Moreover, the external brain anatomy of *C. sardous* does not differ from that of canids included in the group of *Canis*-like taxa [26,27]. However, the reduced number of cochlear turns, which is remarkable in comparison to the number of turns in wild canids [5] and apparently more pronounced than the maximum reduction observed in domestic dogs [40,57], suggests that the Sardinian canid developed peculiar hearing abilities. However, it is difficult to disentangle the causal factors leading to the development of the *C. sardus* apomorphies.

Assuming that the reduced olfactory and visual abilities in mammals of unbalanced insular faunas lacking top predators are manly related to the decreased need to avoid predation, it should be speculated that the changes in hearing ability in this Sardinian predator were possibly related to its hunting behavior and the lifestyle of its preferred prey. Evidence from anatomical characteristics [26,27], dental enamel structure [35,36], and dental microscopic defects (microwear) produced during mastication suggests that the large ochotonid *Prolagus* was the preferred prey of the Sardinian dog *C. sardous*, although it may have occasionally fed on young deer calves (*Praemegaceros cazioti*).

*Prolagus sardus* is the terminal species of the endemic Sardinian ochotonid lineage, characterized by a biphasic evolutionary pattern with a rapid phase following the arrival of the mainland ancestor, giving rise to *Prolagus figaro*, and a relatively slow anagenetic phase developed in relatively stable conditions that originated the larger species *P. sardus.* The latter is the most abundant and frequent species in the Middle and Late Pleistocene Corso-Sardinian faunas in terms of both the number of sites and the abundance of its remains. Such an abundance suggests it was a social, daily species like the extant pika ecotype, inhabiting a variety of vegetated habitats (meadows, steppes, forest, and shrub environments) where they foraged and excavated burrows. The morphology of the *P. sardus* pelvis and limb bones, on the other hand, suggests that the species had powerful hind-limb muscles and was likely adapted to jump and/or climb in rocky environments, such as those characterizing most of the Sardinian landscapes. Moreover, *P. sardus* had a limb maneuverability wider than that shown by most extant leporids [79], although it was likely less adapted to running than other leporids. The Sardinian ochotonid shares these characteristics with mountain pikas rather than meadow-dwelling pikas. Furthermore, detailed morphofunctional studies on the morphofuctional traits of *P. sardus* bone samples from various physiographical contexts may allow detection of the potential presence of more than one pika ecotype in Sardinia.

Therefore, although the acoustic range of *C. sardous* may have been supposedly specialized for identifying the sounds produced by its target prey, imagining what kind of sound frequency was produced by the ochotonid to transmit signals for predator warnings or group communications is a difficult challenge. The Altai pikas, for instance, are known to emit high-frequency (7.31–15.46 kHz) alarm call series and frequency-modulated calls [80], while meadow-dwelling pikas use multiple types of vocalizations, e.g., [81]. Moreover, the impossibility of analyzing laryngeal structures in fossil remains prevent us from verifying the frequencies in the call spectrum of *Cynotherium* and whether it had some similarities with those of the high-frequency and biphonic calls (which are not tied to specific anatomical adaptations of the sound-producing structures) as documented, for instance, in dhole [82].

## 5. Conclusions

This first investigation of the bone labyrinth of *Cynotherium sardous* indicates that the Sardinian canid had reduced turn number and spiral canal length compared to wolf-like dogs and had a cochlear curvature and a basal membrane, suggesting a hearing specialization towards high-frequency sounds with a low limit of 250 Hz and a low echolocalization capability. These results confirm the morphofunctional peculiarities that this intriguing canid developed during its long evolutionary process in an isolated ecosystem characterized by an impoverished and disharmonic fauna, with no top predators but abundant prey, such as *Prolagus sardus*, a very large ochotonid for which *C. sardous* developed a hunting specialization. However, the physiological, ecological, and ethological characteristics of the Sardinian ochotonid are mostly hypothesized and not fully defined. Therefore, we cannot determine whether *C. sardous* detected its prey through echo or had to rely on other senses (such as smell and sight) to locate them and whether its acoustic range was specialized for distinguishing the sounds emitted by its preferred prey. The results obtained confirm the utility of cochlear morphological studies in reconstructing the hearing abilities of the species and providing some suggestions about its ethology, but they fall short of providing any new sound evidence regarding the ecological role of *C. sardous* in the Late Pleistocene Sardinian ecosystem.

## Figures and Tables

**Table 1:** Osteometric data of the cochlea of *Cynotherium sardous* and some mainland carnivore species.

Taxa	Cow	Coh	Con	Col	R_basal_	R_apex_	ρ	Scw	Bmw
*Cynotherium sardous*	4.7	4.1	2.25	25.8	2.35	0.7	3.35	1 turn 1.6 2 turn 0.8 3 turn 0.3	0.4
other Carnivora (mean values)	7.1 *	3.71 *	3.25 **	34.7 *	3.55 *	-	-	-	-

Data are expressed in mm. Abbreviations: Cow, cochlear width that is the maximum diameter of the basal turn of the spiral canal; Coh, cochlear height that is the distance between the plane tangent to the basal turn of the spiral canal and the cochlear apex; Con, number of the turns of spiral canal; Col, length of the spiral canal, imaging the cochlea unrolled; R_basal_, radius of the basal turn; R_apex_, radius of the apical turn; ρ, ratio between the radius of the basal turn and the radius of the apical turn; Scw, width of the spiral canals at different turns; Bmw, width of the basal membrane. * data from [5] refer to 10 felids (*Acinonxy jubatus*, *Leopardus pardalis*, *Leopardus tigrinus*, *Lynx caracal*, *Felis chaus*, *Panthera leo*, *Panthera pardus*, *Puma concolor*, *Prionailurus planiceps*, and *Prionailurus viverrinus*), 4 hyenids (*Crocuta*, *Hyaena*, *Hyaenodon,* and *Proteles cristatus*), 4 viverrids (*Paguma larvata*, *Genetta*, *Arctictis binturong*, and *Viverra tangalunga*), and 1 nandinid (*Nandinia binotata*); ** data from [40] refer to 92 canids (24 *Canis lupus*, 21 prehistoric *Canis familiaris*, 40 modern *Canis familiaris*, and 8 *Canis lupus dingo*).

## Data Availability

All data are included in the results section of the article.
